# Travelling wave analysis in chemotaxis: case of starvation

**DOI:** 10.1186/s40064-016-2507-8

**Published:** 2016-06-29

**Authors:** P. M. Tchepmo Djomegni

**Affiliations:** Department of Mathematical Sciences, University of South Africa, Johannesburg, 0003 South Africa

**Keywords:** Group-invariant solutions, Travelling wave solutions, Metabolism

## Abstract

In this paper we investigate the existence of travelling wave solutions for a chemotaxis model under the scenarios of zero growth and constant growth rate. We use Lie symmetry analysis to generate generalized travelling wave solutions, a wider class of solutions than that obtained from the standard *ansatz*. Unlike previous approaches, we allow for diffusivity and signal degradation. We study the influence of cell growth, diffusivity and signal degradation on the behaviour of the system. We apply realistic boundary conditions to explicitly provide biologically relevant solutions. Our results generalize known results.

## Background

Chemotaxis is orientation (or movement) of an organism in response to chemical signals. Cells, through membrane receptors located at their surface, sense the environment, detect chemicals, and then transfer information to their interior (Berg et al. 2002). Depending on the nature of the information, an enzyme will be produced and will cause cells to respond accordingly (attraction or repulsion). The protein CheY facilitates the transmission of the signal from the chemoreceptors to the flagella motors in *E. coli* (Paul et al. 2010). The phosphorylation of CheY caused by chemorepellents will drive the flagella to rotate clockwise, and the dephosphorylation of CheY caused by chemoattractants will drive counter-clockwise rotation of the flagella (Maki et al. 2000; Eisenbach and Lengeler 2004). Counter-clockwise rotation of the flagella causes the cell to move forward, and clockwise rotation causes the cell to stumble (we note that *E. coli* moves by jumping through the rotation of its flagella).

Progress made in cell biology shows that chemotaxis plays a vital role in reproduction, tissue repair, drug delivery and tumor invasion (Entschladen and Zanker 2002; Friedrich and Jülicher 2007; Schneider et al. 2010; Lajkó et al. 2013; Sahari et al. 2014). In fact, sperm cell motility is directed by chemoattractants resulting from signalling of female reproductive tract (Entschladen and Zanker 2002; Friedrich and Jülicher 2007). In wound healing processes, chemotaxis facilitates the aggregation of immune system cells into site of infection (Schneider et al. 2010). It is also involved in metastasis and atherosclesis states of diseases (Condeelis et al. 2001; Devreotes and Janetopoulos 2003; Gangur et al. 2002; Moore 2001; Murphy 2001). In pharmacology, chemotaxis is involved in drug delivery to the targeted defective area (Lajkó et al. 2013; Sahari et al. 2014). The beauty of the dynamics of chemotaxis is that cells manifest harmonious behaviour, while behaving independently. This was observed independently by Engelmann (1881a, b), Pfeffer (1888) and Beyerinck (1895). With the remarkable work of Adler (1966a, b) in the past fifty years, bacterial chemotaxis became one of the better-documented systems in Biology. Adler (1966a, b, 1975) observed travelling bands of bacteria when he introduced a population of cells (*E. coli*) in a capillary tube accommodating oxygen and an energy source. Two bands of cells were formed; the first band consumed all the oxygen and the second band consumed the residual energy source. Bands were also observed without the adding of the energy source; cells consumed oxygen and excreted a gradient of energy source (Adler 1966b). Bak et al. (1987) noticed that the bands were in a form of a circular ring. The complexity of the geometric patterns caused by chemotaxis cannot be intuitively explained from experiments (Murray 2002). As a result, mathematical modelling approaches have been proposed which have been able to predict the geometric shape of the pattern (Keller and Segel 1970, 1971a, b; Patlak 1953; Scribner et al. 1974; Hillen and Painter 2009).

Keller and Segel (1970, 1971a, b) proposed, for the first time from a population-based perspective, a chemotaxis model (the K–S model) that describes the motion of *slime* and the formation of chemotactic bands of cells. The general form of the K–S model is written as follows:

1 $$\frac{\partial b}{\partial t}=  \nabla \cdot (\mu (s)\nabla b)-\nabla \cdot (b \chi (s) \nabla s),$$

2 $$\frac{\partial s}{\partial t}=  D \nabla ^2 s-k(s) b,$$

where *t* represents the time, *b* is the cell density, *s* the concentration of the critical substrate, $\chi (s)$ the chemotactic sensitivity, *k*(*s*) the consumption rate of substrates per cell, and $\mu (s)$ and *D* the diffusion coefficient of the bacteria and the substrates, respectively. Note that cell proliferation was ignored in the K–S model. A singularity in the chemotactic sensitivity was required to produce travelling wave solutions (Keller and Segel 1970, 1971a; Scribner et al. 1974). Such a hypothesis is problematic, given that it can cause the bands to move with unbounded velocity (we note that the speed of the band should not be larger that the speed of a single cell) (Xue et al. 2011). This unnecessary restriction can be overcome by the consideration of other relevant factors. It was shown, for instance, that adding of logistic growth terms can lead to travelling wave solutions (with non-singular sensitivity) (Nadin et al. 2008). In the case of logarithmic chemotactic sensitivity, Wang (2013) demonstrated that the adding of substrates degradation does not produce travelling wave solutions.

From the cell-based perspective, Patlak (1953) proposed the first model for chemotaxis to depict the random walk process of a particle with external bias and persistence of direction. This model was later improved by Alt (1980) and Othmer et al. (1988). Recently, Xue et al. (2011) formulated a model which takes into account the interaction between two substrates (nutrients and attractants). What is remarkable about their model is that it can be applied in a variety of biological situations, including population dynamics to describe the competition between two species from a microscopic level (the individual species behaviour). Variables describing intracellular processes such as metabolism and transduction of the signal were explicitly represented in the Xue et al. ’s (2011) model. Travelling wave solutions with a unique wave speed were demonstrated in the scenario of zero growth, without requiring a singularity in the chemotactic sensitivity. We Tchepmo Djomegni and Govinder (2015b, 2016) extended these results by allowing for diffusivity and cell proliferation, and provided explicit solutions for the first time. Franz et al. (2014) studied the case of starvation. They assumed that cells consume chemoattractants only (which do not diffuse over the space), and considered a non constant growth of bacteria. They proved the existence of travelling wave solutions in the case of no chemotaxis. It has been proved that the parabolic limit of the microscopic model is the Keller–Segel model (Lui and Wang 2010).

In this paper, we will be looking at the individual behaviour of cells to understand the convergence and harmonization of their motion. The aggregation and movement (with constant speed) of cells are the centre of our study. We will focus on the case of low presence (or absence) of nutrients as the formation of bands of cells was observed in this situation (Adler 1966b; Brenner et al. 1998). The existence of travelling wave solutions will be investigated. Unlike previous approaches, we will allow for diffusivity, and will account for signal degradation and constant cell growth. We will also study the impact of microscale parameters (such as cell growth rate, cell unbiased turning rate and cell speed) on the macroscopic behaviour of the system.

We introduce the model in “Reduced model and analysis” section. As symmetry analysis has proven to be very effective in finding useful solutions to PDEs (Clarkson 1995), we utilise that approach for our system of PDEs. We generate a class of invariants that lead to generalized travelling wave solutions. In some cases, we utilise dynamical systems analysis to further investigate the behaviour of the solutions. (This confirms our previous findings on the interplays between group theory and dynamical systems analysis Tchepmo Djomegni and Govinder 2014.) Realistic initial and boundary conditions are then applied to obtain relevant solutions. We discuss our results in “Discussion” section.

## Reduced model and analysis

The model emanates from previous experiments (Blat and Eisenbach 1995; Budrene and Berg 1991, 1995; Woodward et al. 1995) in which bacteria (*E. coli*) consume nutrients and excrete a signal gradient, then aggregate in different patterns formed in response to this gradient. We are concerned with the case of limited resources (low presence or absence of nutrients). In this scenario cells consume the excreted signal only. A set of chemical processes occur within the cells to enable them to survive and respond to their surroundings. Xue et al. (2011) developed a model to describe the intracellular metabolism, written as follows:

3 $$\frac{d z_{1}}{d t}=\frac{F(x,t)-z_{1}}{t_{f}}, \quad \frac{d z_{2}}{d t}=\frac{z_{1}-z_{2}}{t_{m}},$$

where $z=(z_{1},\,z_{2})$ depicts the cellular metabolism, *F*(*x*, *t*) is the concentration of nutrients, and $t_{f}$ and $t_{m}$ are the characteristic time scales for the production of the immediate variables $z_{1}$ and $z_{2}$, respectively. In the above description, it is assumed that after consumption of succinate *F*(*x*, *t*) a variable $z_{1}$ is involved to facilitate the production of the signal *S*(*x*, *t*) via the pathway

4 $$F \rightarrow\,z_{1} \rightarrow\,S.$$

The low level of nutrients will cause $z_{1}$ to catalytically influence the production of a starving variable $z_{2}$, via the metabolic pathway

5 $$\phi \xrightarrow {z_{1}} z_{2} \rightarrow \phi , $$

where $\phi$ stands for the reactants/products assumed to be in excess (Xue et al. 2011).

We overlook the explicit representation of the variable $z_{1}$ (given that $t_{f} \lll t_{m}$), and we assume fast signal transduction (given that the adaptation time of the signal transduction $t_{a} \lll t_{m}$ Xue et al. 2011). Then the distribution of the cells can be described in one-dimensional space as follows:

6 $$\frac{\partial p^{+}}{\partial t}+s \frac{\partial p^{+}}{\partial x}+\frac{\partial }{\partial z}\left( \frac{F-z}{t_{m}} p^{+} \right) =-\lambda \left( -\frac{\partial S}{\partial x} \right) p^{+}+\lambda \left( \frac{\partial S}{\partial x} \right) p^{-}+h(S) p^{+},$$

7 $$\frac{\partial p^{-}}{\partial t}-s \frac{\partial p^{-}}{\partial x}+\frac{\partial }{\partial z}\left( \frac{F-z}{t_{m}} p^{-} \right) =\lambda \left( -\frac{\partial S}{\partial x} \right) p^{+}-\lambda \left( \frac{\partial S}{\partial x} \right) p^{-}+h(S) p^{-},$$

where $p^{\pm }(x,z,t)$ is the density of cells at the position *x*, the internal state $z=z_{2}$ and time *t*, moving with constant speed ${\pm}s$, $\lambda$ and *h* are the turning rate function and the proliferation rate of the cells, respectively. We will consider the following turning rate function (Xue et al. 2011):

8 $$\lambda \left( \xi \right) =\lambda _{0} \left( 1+\xi \chi (\xi ) \right),$$

where $\lambda _{0}$ is the unbiased turning rate ($\lambda _{0}>0$) and $\chi (\xi ) =\left( k+| \xi |\right) ^{-1}$ is the chemotactic sensitivity function, with *k* being the sensitivity coefficient. By letting

9 $$n(x,t)=\int _{{\mathbb {R}}} (p^{+}(x,z,t)+p^{-}(x,z,t))dz, \quad j(x,t)=\int _{{\mathbb {R}}} s (p^{+}(x,z,t)-p^{-}(x,z,t))dz,$$

10 $$\lambda ^{1} \left( \frac{\partial S}{\partial x} \right) =\lambda \left( \frac{\partial S}{\partial x} \right) -\lambda \left( -\frac{\partial S}{\partial x} \right) , \quad \lambda ^{2} \left( \frac{\partial S}{\partial x} \right) =\lambda \left( \frac{\partial S}{\partial x} \right) +\lambda \left( -\frac{\partial S}{\partial x} \right) =2\lambda _{0},$$

and integrating (6–7) over *z*, one can transform (6–7) into

11 $$\frac{\partial n}{\partial t}+\frac{\partial j}{\partial x}=  h(S) n,$$

12 $$\frac{\partial j}{\partial t}+s^2\frac{\partial n}{\partial x}= s \lambda ^{1} n-2\lambda _{0} j+h(S) j.$$

The functions *n*(*x*, *t*) and *j*(*x*, *t*) are respectively the macroscopic cell density and the flux.

The equations describing the distribution of the signal can be given by

13 $$\frac{\partial S}{\partial t }=D_{S} \frac{\partial ^2 S}{\partial x^2}-\alpha S n-\gamma S,$$

with $\alpha$ and $\gamma$ standing for the consumption rate and degradation rate of the aspartate, respectively.

Note that the case $k=0$ corresponds to unbounded sensitivity to the signal (Xue et al. 2011). As a result, the function $\lambda ^{1}$ becomes the switch function

14 $$\lambda ^{1} \left( \frac{\partial S}{\partial x} \right) = {\left\{ \begin{array}{ll} -2 \lambda _{0}, &\quad \partial S/\partial x < 0, \\ 0, &\quad \partial S/\partial x =0, \\ 2 \lambda _{0}, &\quad \partial S/\partial x > 0. \end{array}\right.} $$

The situation of no chemotaxis (i.e., $\chi =0$) takes place when $k \rightarrow \infty$, and we have $\lambda ^{1}=0$. Here, cells are not sensitive to the signal. Our analysis will focus on these two limiting cases of unbounded sensitivity to the signal and no chemotaxis. Thought it is very hard to obtain travelling wave solutions technically if *k* varies, it has numerically been shown that increasing chemotactic responses results to an increase in the wave propagation (Franz et al. 2014).

The system (11–13) was analysed in the case of no chemotaxis by Franz et al. (2014). They assumed that $D_{S}=\gamma =0$, and *h*(*S*) is a linear function of *S*. Due to the complexity of the system, diffusivity has always been ignored in the mathematical analysis. It is important to note that diffusivity plays a stabilizing role in the behaviour of the system (Rosen 1977). As a result, in our analysis, we will allow for diffusivity, and will investigate the existence of travelling wave solutions under zero growth and constant growth scenarios [We note that demonstrating the existence of traveling wave solutions is equivalent to demonstrating the existence of solutions to (11–13) (Lui and Wang 2010)]. The impact of the growth rate in the behaviour of the solutions will be explored.

### Lie symmetry analysis

Examining the interplay between group theory and stability analysis, we found (Tchepmo Djomegni and Govinder 2016, 2014) that the Lie symmetry analysis can generate new types of solutions (unlike the standard travelling wave ansatz) that play a significant role in the stability of the system. A partial differential equation of order *n*,

15 $$E(x,y,\partial y,\ldots ,\partial ^{n} y)=0, $$

with $$(x,y(x))\in {\mathbb {R}}^{N} \times {\mathbb {R}}^{M}$$, possesses

16 $$G= \sum _{i=1}^{N} \xi _{i}(x,y) \partial _{ x_{i}}+ \sum _{j=1}^{M} \eta _{j}(x,y)\partial _{ y_{j}},$$

as a symmetry if (Bluman and Anco 2002)

17 $$ G^{[n]}E \mid _{E=0} \,=\,0,$$

where $\xi _{i}(x,y)$ and $\eta _{j}(x,y)$ are the infinitesimals of the Lie group of invariant transformations of (15), and $G^{[n]}$ is the *nth* extension of *G* (Bluman and Anco 2002). The operator *G* in (16) helps to reduce the number of independent variables and the order of equations. We note that a linear combination of symmetries to an equation is also a symmetry to that equation. The expression of $G^{[n]}$ can be obtained in Bluman and Anco (2002).

In the case of (11–13), we have

18 $$ G= \xi _{1}(t,x,n,j,S) \partial _{ t}\,+\,\xi _{2}(t,x,n,j,S) \partial _{ x}\,+\, \eta _{1}(t,x,n,j,S) \partial _{ n}\,+\,\eta _{2}(t,x,n,j,S) \partial _{ j}\,+\,\eta _{3}(t,x,n,j,S) \partial _{ S}.$$

Applying the second extension $G^{[2]}$ of (18) to the Eqs. (11), (12) and (13) (with $\lambda ^{1}$ treated as a constant), we obtain the symmetries

19 $$G_{1}= \partial _{t},{\quad} G_{2}= \partial _{x}, {\quad}G_{3}={\rm e}^{-2\lambda _{0}t}\partial _{j},{\quad}G_{4}=S\partial _{S},{\quad} G_{5}= \frac{-s \lambda ^{1}}{2\alpha \lambda _{0}}\partial _{j}-\frac{1}{\alpha }\partial _{n}+t S \partial _{S},$$

20 $$G_{6}=   \left( -\frac{s t \lambda ^{1}+2x\lambda _{0}}{ 2\lambda _{0}}\partial _{j}-\frac{1}{2\lambda _{0}}\partial _{n}-\frac{\alpha }{\lambda ^{2}} S \partial _{S} \right) {\rm e}^{-2\lambda _{0}t}, $$

in the case of zero growth (i.e., $h(S)=0$). In the case of constant growth (with $h(S)=\alpha _{0}$), we have

21 $$G_{1}= \partial _{t}, {\quad}G_{2}= \partial _{x}, {\quad} G_{3}={\rm e}^{(\lambda _{0}-2\lambda _{0})t}\partial _{j}, {\quad}G_{4}=S\partial _{S},  {\quad}G_{5}=\left( \frac{-s \lambda ^{1}}{2\alpha \lambda _{0}}\partial _{j}-\frac{1}{\alpha }\partial _{n}+\frac{1}{\alpha } S \partial _{S}\right) {\rm e}^{\alpha _{0}t}, $$

22 $$G_{6}= \left( -\frac{s t \lambda ^{1}+2x\lambda _{0}}{ \alpha }\partial _{j}-\frac{1}{\alpha }\partial _{n}-\frac{1}{\alpha _{0}-2\lambda _{0}} S \partial _{S} \right) {\rm e}^{(\alpha _{0}-2\lambda _{0})t},$$

for $$\alpha _{0}\ne 2\lambda _{0}$$, and

23 $$G_{1}= \partial _{t}, {\quad} G_{2}= \partial _{x}, {\quad} G_{3}=\partial _{j}, {\quad}G_{4}=S\partial _{S}, {\quad} G_{5}=-\frac{1}{\alpha }\partial _{n}+t S \partial _{S},$$

24 $$G_{6}= \frac{x}{ \alpha }\partial _{j}+\frac{1}{2\alpha \lambda _{0}}{\rm e}^{-2\lambda _{0} t} \partial _{n}+\frac{1}{4\lambda _{0}^2} S {\rm e}^{-2\lambda _{0} t} \partial _{S}, $$

for $\alpha _{0}=2\lambda _{0}$. We let

25 $$G=G_{1}+c G_{2}+c_{1} G_{3}+c_{2} G_{4}+c_{3} G_{5}+c_{4} G_{6}, $$

where the coefficients *c*, $c_{1}$, $c_{2}$ and $c_{3}$ are constants.

In the case of zero growth, using (19–20), the characteristic equations associated with *G* are (refer to Bluman and Anco 2002)

26 $$\dfrac{dt}{1}=\dfrac{dx}{c}=\dfrac{2\alpha \lambda _{0} {\rm e}^{2\lambda _{0} t} dj}{2\alpha \lambda _{0} c_{1} -\alpha c_{4} (st\lambda ^{1}+2x \lambda _{0})}=\dfrac{-2\alpha \lambda _{0} dn}{2\lambda _{0} c_{3}-\alpha c_{4} {\rm e}^{-2\lambda _{0} t}}=\dfrac{4\lambda _{0}^{2} dS}{(4\lambda _{0}^2 c_{2}+4\lambda _{0}^2 c_{3} t-\alpha c_{4} {\rm e}^{-2\lambda _{0} t})S}.$$

These lead to the new invariants

27 $$u= x-c t, $$

28 $$j= J(u)+\dfrac{{\rm e}^{-2\lambda _{0}t}(4\lambda _{0}^2 c_{4} u-4c_{1} \lambda _{0}^2+c_{4} (s \lambda ^{1}+2c \lambda _{0})(1+2\lambda _{0}t))}{8\lambda _{0}^3},$$

29 $$n= N(u)-\frac{c_{3} t}{\alpha }-\frac{c_{4} {\rm e}^{-2\lambda _{0}t}}{4\lambda _{0}^2},$$

30 $$S= S_{1}(u)\exp \left( c_{2} t+\frac{1}{2} c_{3} t^2+\frac{\alpha c_{4}{\rm e}^{-2\lambda _{0}t}}{8\lambda _{0}^{3}}\right) .$$

We note from (27), that travelling wave solutions can exist, with *c* being the speed of the wave. As we require that solutions should not blow up as $x \rightarrow \pm \infty$ or $t \rightarrow \infty$, we take $c_{3}=c_{4}=0$. Therefore, from the definition of the flux *j* (see (9)), we obtain $c_{1}=0$.

In the case of constant growth with $\alpha _{0} \ne 2\lambda _{0}$, the characteristic equations associated with G, now using (21–22), are

31 $$\begin{aligned} \dfrac{dt}{1}=\dfrac{dx}{c} &=  \dfrac{2\alpha \lambda _{0} {\rm e}^{(2\lambda _{0}-\alpha _{0}) t} dj}{2\alpha \lambda _{0} c_{1}-s c_{3} \lambda ^{1} {\rm e}^{2\lambda _{0} t} -2\lambda _{0} c_{4} (st\lambda ^{1}+2x \lambda _{0})}=\dfrac{-\alpha dn}{ c_{3} {\rm e}^{\alpha _{0}t}+ c_{4} {\rm e}^{(\alpha _{0}-2\lambda _{0}) t}}  \\&= \dfrac{\alpha _{0} (\alpha _{0}- 2\lambda _{0}) dS}{\left( \alpha _{0} (\alpha _{0}- 2\lambda _{0})c_{2}+(\alpha _{0}- 2\lambda _{0}) c_{3} {\rm e}^{\alpha _{0}t}+\alpha _{0} c_{4} {\rm e}^{(\alpha _{0}-2\lambda _{0})t})\right) S}, \end{aligned}$$

and lead to the invariants

32 $$u= x-c t, $$

33 $$\begin{aligned} j&= J(u)+\dfrac{{\rm e}^{(\alpha _{0}-2\lambda _{0})t}\left( s \lambda ^{1} c_{3} (\alpha _{0}-4\lambda _{0})^2 {\rm e}^{2\lambda _{0}t}-2\alpha \alpha _{0} c_{1} \lambda _{0} (\alpha _{0}-2\lambda _{0})\right) }{2\alpha \alpha _{0} (\alpha _{0}-2\lambda _{0})^2 \lambda _{0}}  \\&\quad +\frac{2\alpha _{0} \lambda _{0} c_{4} (-1+t(\alpha _{0}-2\lambda _{0})(s \lambda ^{1}+2c \lambda _{0})){\rm e}^{(\alpha _{0}-2\lambda _{0})t}-c_{4} {\rm e}^{(\alpha _{0}-2\lambda _{0})t} }{\alpha (\alpha _{0}-2\lambda _{0})}, \end{aligned}$$

34 $$n= N(u)-\frac{ (\alpha _{0}-2\lambda _{0}) c_{3} {\rm e}^{\alpha _{0} t}+\alpha _{0} c_{4} {\rm e}^{(\alpha _{0}-2\lambda _{0})t}}{\alpha \alpha _{0} (\alpha _{0}-2\lambda _{0})},$$

35 $$S= S_{1}(u)\exp \left( \frac{\alpha _{0}^2 c_{4} {\rm e}^{(\alpha _{0}-2\lambda _{0})t}+ (\alpha _{0}-2\lambda _{0})^2 c_{3} {\rm e}^{\alpha _{0}t} +\alpha _{0}^2 (\alpha _{0}-2\lambda _{0})^2 c_{2} t }{\alpha _{0}^2 (\alpha _{0}-2\lambda _{0})^2} \right) . $$

As before, we take $c_{1}=c_{3}=c_{4}=0$ for physically viable (bounded) solutions. The same conditions apply in the case of $\alpha _{0}=2\lambda _{0}$. As a result in our analysis we use the following invariants:

36 $$u= x-c t, $$

37 $$j= J(u), $$

38 $$ n=   N(u), $$

39 $$ S= S_{1}(u){\rm e}^{c_{2} t}, $$

in all cases. We note that (37–38) are generalized travelling wave solutions (Polyanin and Zaitsev 2004; Tchepmo Djomegni and Govinder 2016). The coefficients $c_{2}$ can produce to damped (when $c_{2}<0$) or growing (when $c_{2}>0$) solutions. The case $c_{2}=0$ lead to the standard ansatz travelling wave solutions. Then the system (11–13) can be rewritten in term of the new invariants as follows:

40 $$(s^2-c^2) N^{\prime}= (c h(S)+s \lambda ^{1}) N+(h(S)-2\lambda _{0})J,$$

41 $$(s^2-c^2) J^{\prime}= (s^2 h(S)+cs \lambda ^{1}) N+c(h(S)-2\lambda _{0})J, $$

42 $$ -c S_{1}^{\prime}=  D_{S} S_{1}^{\prime\prime}-(\alpha N+\gamma +c_{2}) S_{1}, $$

where the superscript ′ stands for the total derivative with respect to *u*. When $c=s$, (40–42) can be reduced to a system of two equations in three unknowns. Choosing *J*(*u*) and $S_{1}(u)$ to depend on *N*(*u*), we can demonstrate travelling wave solutions for a constant distribution of *N*(*u*) [by simply solving the second order ODE with constant coefficients (42)]. The analysis for the case of Poisson distribution (or normal distribution via asymptotic analysis) of *N*(*u*) is similar to the analysis when $c\ne s$. We will focus in the rest of this work on the case of $c\ne s$ (with $0<c<s$). Inspired by the numerical investigations of Xue et al. (2011), we will be looking for solutions admitting a single peak of *S*. We note that this restriction is less important when $k\rightarrow \infty$. Hence we assume $S_{1} \in Y_{S}$, where

43 $$\begin{aligned}Y_{S}=  \lbrace & f \in {\mathbb {C}}^{1}({\mathbb {R}}); f(u)\,\hbox {is\,monotonically\,increasing\,for}\,u<0  \\&\hbox {and\,decreasing\,for}\,u>0 \rbrace. \end{aligned}$$

In our context, travelling wave solutions *n*(*x*, *t*) and *S*(*x*, *t*) must be positive, continuous and bounded, with $S_{1} \in Y_{S}$.

### Case of zero growth

Here $h(S)=0$. Assuming that *N*(*u*) and *J*(*u*) decay to zero as $u \rightarrow \infty$, (40–42) can be reduced to

44 $$J= c N, $$

45 $$(s^2-c^2) N^{\prime}= (s \lambda ^{1}-2c \lambda _{0}) N, $$

46 $$-c S_{1}^{\prime}= D_{S} S_{1}^{\prime\prime}-(\alpha N+\gamma +c_{2}) S_{1}.$$

In the case of high chemotactic sensitivity (the limiting case $k \rightarrow 0$), the turning rate function becomes a switch function and corresponds to unbounded sensitivity to the signal (Xue et al. 2011). For $S_{1} \in Y_{S}$, the solution *n*(*x*, *t*) is given by

47 $$n(x,t)=N(u)= {\left\{ \begin{array}{ll} N(0) {\rm e}^{\sigma _{1}u}, &{\quad} u < 0, \\ N(0) {\rm e}^{-\sigma _{2}u}, &{\quad} u \ge 0, \end{array}\right. }$$

where $\sigma _{1}=2 \lambda _{0}/(s+c)$ and $\sigma _{2}=2 \lambda _{0}/(s-c)$. Here, the total cell population is given by $T=sN(0)/\lambda _{0}$ (obtained by integrating *N*(*u*) over the whole line ${\mathbb {R}}$).

We assume $D_{S} =0$, then $S_{1}(u)$ is positive and continuous. When $\gamma +c_{2} \ge 0$, $S_{1}(u)$ is monotonically increasing ($S^{\prime}_{1}(u)>0$). As a result, $S_{1} \in Y_{S}$ cannot hold. Since $S^{\prime}_{1}(u)$ is continuous, for $S_{1}$ to hold in $Y_{S}$ when $\gamma +c_{2}<0$, it is necessary that zero must be the only extremum point of $S_{1}(u)$ (the maximum), with $\alpha N(0)=-(\gamma +c_{2})$ (because $S_{1}^{\prime}(0)=0$). Then, for $u<0$,

48 $$cS_{1}^{\prime}(u)=(\alpha N(u)+\gamma +c_{2})S_{1}(u)=\alpha N(0) ({\rm e}^{\sigma _{1}u}-1)S_{1}(u)<0. $$

Again $S_{1} \notin Y_{S}$. Non-diffusing travelling wave solutions with a single peak of *S* do not exist.

In the case of diffusivity, substituting (47) into (46) and integrating, we obtain

49 $$S_{1}(u)= {\left\{ \begin{array}{ll} \left[ c_{1}^{1} I_{k_{1}}\left( \alpha _{1} {\rm e}^{(\sigma _{1}/2) u} \right) +c_{2}^{1} K_{k_{1}}\left( \alpha _{1} {\rm e}^{(\sigma _{1}/2) u} \right) \right] {\rm e}^{-(c/(2D_{S})) u}, &{\quad} u < 0, \\ \left[ c_{1}^{2} I_{k_{2}}\left( \alpha _{2} {\rm e}^{- (\sigma _{2}/2) u} \right) +c_{2}^{2} K_{k_{2}}\left( \alpha _{2} {\rm e}^{-(\sigma _{2}/2) u} \right) \right] {\rm e}^{-(c/(2D_{S})) u}, &{\quad} u \ge 0, \end{array}\right. }$$

where $k_{i}=\sqrt{c^2+4D_{S}(\gamma +c_{2})}/(D_{S} \sigma _{i})$, $\alpha _{i}=\sqrt{4 \alpha D_{S} N(0)}/(D_{S} \sigma _{i})$, the coefficients $c_{j}^{i}$ are constants of integration, and the functions $I_{k_{i}}(v)$ and $K_{k_{i}}(v)$ are the two linearly independent solutions to the modified Bessel’s equation.

#### **Proposition 1**

*For* $-\gamma <c_{2}\le 0$, *the function* $S(x,t)=S_{1}(u) {\rm e}^{c_{2}t}$, *where* $S_{1}(u)$ *is given by (* 49 *), is bounded if and only if* $c_{2}^{1}=c_{2}^{2}=0$.

#### *Proof*

We choose $c_{2}\le 0$ in order to produce damped solutions. We note that the functions $I_{k_{i}}(v)$ and $K_{k_{i}}(v)$ given in (49) are continuous and positive (McLachlan 1955; Olver et al. 2010).

We assume $u=x-c t<0$. Then $I_{k_{1}}\left( \alpha _{1} {\rm e}^{(\sigma _{1}/2)u}\right)$ converges to zero and $K_{k_{1}}\left( \alpha _{1} {\rm e}^{(\sigma _{1}/2)u}\right)$ diverges, as $u \rightarrow -\infty$ (McLachlan 1955; Olver et al. 2010). If $c_{2}^{1} \ne 0$, *S*(*x*, *t*) blows up as $x \rightarrow -\infty$. However, if $c_{2}^{1}=0$, as $u \rightarrow -\infty$,

50 $$I_{k_{1}}\left( \alpha _{1} {\rm e}^{(\sigma _{1}/2)u}\right) \approx \frac{(\alpha _{1}/2)^{k_{1}}}{{\varGamma }(1+k_{1})} {\rm e}^{\frac{\sigma _{1} k_{1}}{2}u} \approx \frac{(\alpha _{1}/2)^{k_{1}}}{{\varGamma }(1+k_{1})} {\rm e}^{\frac{\sqrt{c^2+4 D_{S}(\gamma +c_{2})}}{2 D_{S}}u}.$$

This implies that

51 $$S_{1}(u) \approx \frac{c_{1}^{1}(\alpha _{1}/2)^{k_{1}}}{{\varGamma }(1+k_{1})} {\rm e}^{\frac{\sqrt{c^2+4 D_{S}(\gamma +c_{2})}-c}{2 D_{S}}u}, $$

and converges to zero as $u \rightarrow -\infty$ (provided $\gamma +c_{2}>0$). Then *S*(*x*, *t*) converges to zero as $x \rightarrow \pm \infty$ or $t \rightarrow \infty$ (with $x-ct<0$).

Now we assume $u\ge 0$. As $u \rightarrow \infty$,

52 $$K_{k_{2}}\left( \alpha _{2} {\rm e}^{-(\sigma _{2}/2)u}\right) \approx \frac{{\varGamma }(k_{2})}{2(\alpha _{2}/2)^{k_{2}}} {\rm e}^{\frac{\sigma _{2} k_{2}}{2}u} \approx \frac{{\varGamma }(k_{2})}{2(\alpha _{2}/2)^{k_{2}}} \hbox{e}^{\frac{\sqrt{c^2+4 D_{S}(\gamma +c_{2})}}{2 D_{S}}u}, $$

which implies that

53 $$K_{k_{2}}\left( \alpha _{2} \hbox{e}^{-(\sigma _{2}/2)u}\right) \hbox{e}^{-\frac{c}{2 D_{S}}u} \approx \frac{{\varGamma }(k_{2})}{2(\alpha _{2}/2)^{k_{2}}} \hbox{e}^{\frac{\sqrt{c^2+4 D_{S}(\gamma +c_{2})}-c}{2 D_{S}}u}, $$

as $u \rightarrow \infty$. Therefore, if $c_{2}^{2} \ne 0$, *S*(*x*, *t*) will blow up as $x \rightarrow \infty$ (with *t* fixed). However, if $c_{2}^{2}=0$, $S_{1}(u)$ converges to zero as $u \rightarrow \infty$ (given that $I_{k_{2}}\left( \alpha _{2} \hbox{e}^{-(\sigma _{2}/2)u}\right)$ converges to zero $u \rightarrow \infty$). As a result, *S*(*x*, *t*) converges to zero as $x \rightarrow \infty$ or $t \rightarrow \infty$.

Given that the only point in which $S_{1}(u)$ may not be continuous is zero, and that $S_{1}(0)$ exists, then the convergence of *S*(*x*, *t*) at the boundaries guarantees the boundedness of *S*(*x*, *t*).

#### **Theorem 1**

*For* $D_{S} \ne 0$ *and* $-\gamma <c_{2} \le 0$, *travelling wave solutions for the system (* 11–13) (*with* $h(S)=0$) *exist and are explicitly given by* (47), (44) *and* $S(x,t)=S_{1}(u)\hbox{e}^{c_{2}t}$, *where*

54 $$S_{1}(u)= {\left\{ \begin{array}{ll} c_{1}^{1} I_{k_{1}}\left( \alpha _{1} \hbox{e}^{(\sigma _{1}/2) u} \right) \hbox{e}^{-(c/(2D_{S})) u}, & u < 0, \\ c_{1}^{2}I_{k_{2}}\left( \alpha _{2} \hbox{e}^{- (\sigma _{2}/2) u} \right) \hbox{e}^{-(c/(2D_{S})) u}, & u \ge 0, \end{array}\right. }$$

*with* $$c_{1}^{1}=S_{1}(0)/I_{k_{1}}(\alpha _{1})$$ *and* $$c_{1}^{2}=S_{1}(0)/I_{k_{2}}(\alpha _{2})$$.

#### *Proof*

Invoking Proposition 1, we note that *n*(*x*, *t*) and *S*(*x*, *t*) are positive, continuous and bounded, and the function $\hbox{e}^{-(c/(2D_{S}))u}$ is monotonically decreasing. We only need to show that $S_{1} \in Y_{S}$.

When $u\ge 0$, the function $I_{k_{2}}\left( \alpha _{2} \hbox{e}^{-(\sigma _{2}/2) u} \right)$ is decreasing. Therefore, $S_{1}(u)$ is also monotonically decreasing, and converges to zero as $u \rightarrow \infty$.

When $u<0$, the function $I_{k_{1}}\left( \alpha _{1} \hbox{e}^{(\sigma _{1}/2) u} \right)$ is increasing, and from (50), we have

55 $$f(u)=\frac{(\alpha _{1}/2)^{k_{1}}}{{\varGamma }(1+k_{1})} \hbox{e}^{\frac{\sqrt{c^2+4 D_{S}(\gamma +c_{2})}}{2 D_{S}}u} \le I_{k_{1}}\left( \alpha _{1} \hbox{e}^{(\sigma _{1}/2) u} \right) . $$

Given that the function $f(u) \hbox{e}^{-(c/(2D_{S})u}$ is monotonically increasing, then $S_{1}(u)$ is also monotonically increasing. Consequently, $S_{1} \in Y_{S}$.

**Fig. 1:**
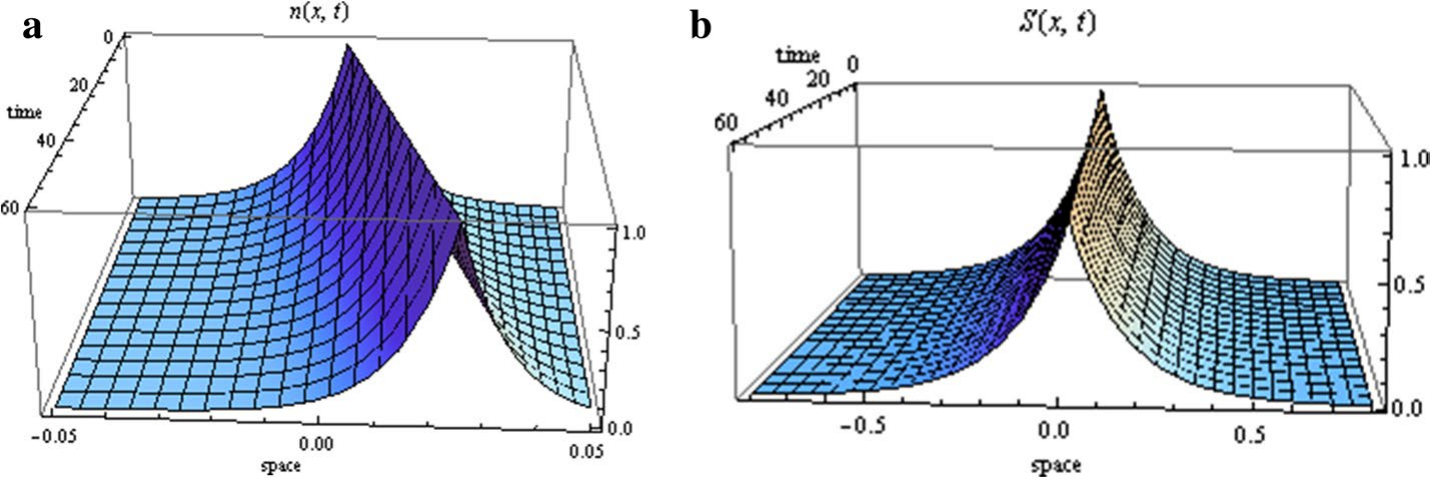
Distribution of cells (**a**) and signal (**b**) in the case of zero growth ($\lambda _{0}=1/sec$, $N(0)=1$ and $S_{1}(0)=1$)

An example of the travelling wave solutions indicated in Theorem 1 are illustrated in Fig. 1 with $s=20\,\upmu \hbox{m/s}$, $D_{S}=10^{-5}\hbox {cm}^2/\hbox {s}$, $\alpha =1000/\hbox {h}$, $\gamma =0.05/\hbox {s}$, $c=1.5\,\hbox {mm}/\hbox {h}$ (Xue et al. 2011) (these parameter values will be used in all future plots).

In the case of no chemotaxis (i.e., $\chi =0$), we have $\lambda ^{1}=0$. Then from (45), *N*(*u*) blows up as $u\rightarrow -\infty$; cells can only aggregate in the half plane $u\ge 0$. Travelling wave solutions satisfying $S_{1} \in Y_{S}$ do not exist. This is consistent with Xue et al. (2011). However, if we relax the assumption on $S_{1}$, we can demonstrate travelling wave solutions.

#### **Theorem 2**

*In the absence of chemotaxis, generalized travelling wave solutions for the system (* 11–13 *) exist (without the restriction* $S \in Y_{S}$ *) and are explicitly given in the case of non diffusivity (* $D_{S}=0$ *) by*

56 $$n(x,t)=N(u)= N(0) \hbox{e}^{-\sigma _{0} u}, \quad j(x,t)=J(u)=c N(0) \hbox{e}^{-\sigma _{0} u}, $$

*and* $$S(x,t)=S_{1}(u)\hbox{e}^{c_{2} t}$$ *, where*

57 $$S_{1}(u)=S_{1}(0) \exp \left( \frac{\alpha N(0)}{c \sigma _{0}}(1-\hbox{e}^{-\sigma _{0} u}) +\frac{\gamma +c_{2}}{c}u \right) ,$$

*with* $u \ge 0$, $\sigma _{0}=2\lambda _{0} c/(s^2-c^2)$ *and* $c_{2}\le -\gamma$, *and in the case of diffusivity* ($D_{S}\ne 0$) *by*

58 $$S_{1}(u)= S_{1}(0) I_{k_{0}}\left( \alpha _{0,0} \hbox{e}^{-(\sigma _{0}/2)u}\right) \hbox{e}^{-(c/(2D_{S}))u}/I_{k_{0}}(\alpha _{0,0}),$$

*with* $$u\ge 0$$, $$k_{0}=\sqrt{c^2+4D_{S}(\gamma +c_{2})}/(D_{S} \sigma _{0})$$, $$\alpha _{0,0}=\sqrt{4 \alpha D_{S} N(0)}/(D_{S} \sigma _{0})$$ *and* $$-\gamma < c_{2}\le 0$$.□

**Fig. 2:**
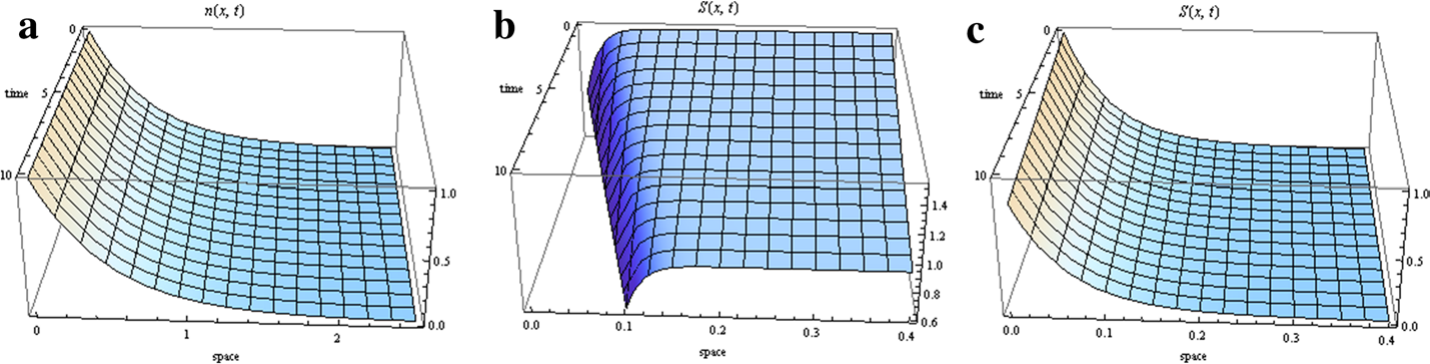
Distribution of cells in the absence of chemotaxis under zero growth (we used $c=10\,\upmu \hbox {m/s}$ and $c_{2}=-\gamma =-0.05/\hbox {s}$, for $D_{S}=0$, and $c=1.5\,\hbox {mm/h}$ and $c_{2}=-0.02/\hbox {s}$, for $D_{S}\ne 0$). **a** Cells, **b** aspartate ($D=0$), **c** aspartate ($D_s\ne 0$)

The proof follows the same procedure as Theorem 1. The solutions are illustrated in Fig. 2.

### Case of constant growth $h(S)=\alpha _{0}$

#### No chemotaxis ($k \rightarrow \infty$)

Here, $\chi =0$. As a result, $\lambda ^{1}=0$. Then (40–42) becomes

59 $$N^{\prime}= f_{1} N+g_{1} J, $$

60 $$J^{\prime}= f_{2} N+c g_{1} J,$$

61 $$-c S^{\prime}_{1}= D_{S} S^{\prime\prime}_{1} -(\alpha N+\gamma +c_{2}) S_{1}, $$

where

62 $$f_{1}=\frac{c \alpha _{0}}{s^2-c^2}, \quad g_{1}=\frac{\alpha _{0}-2\lambda _{0}}{s^2-c^2}, \quad f_{2}=\frac{s^2 \alpha _{0}}{s^2-c^2}. $$

When $\alpha _{0}=2 \lambda _{0}$ (i.e., $g_{1}=0$), *N*(*u*) blows up as $u \rightarrow \infty$. In this situation, cells can only move in the half plane $u \le 0$. As a result, $S_{1}$ cannot hold in $Y_{S}$.

Assuming $\alpha _{0} \ne 2 \lambda _{0}$, *N*(*u*) and *J*(*u*) are given by

63 $$\left( \begin{array}{l} N(u) \\ J(u) \end{array}\right) = C_{1} \left( \begin{array}{l} \gamma _{3} \\ 1 \end{array}\right) \hbox{e}^{\lambda _{1} u}+C_{2} \left( \begin{array}{l} \gamma _{4} \\ 1 \end{array}\right) \hbox{e}^{\lambda _{2} u}, $$

where

64 $$\begin{aligned} \lambda _{1}&= \frac{-c(\lambda _{0}-\alpha _{0})+\sqrt{\alpha _{0}^2 s^2-2 \lambda _{0} \alpha _{0}s^2+c^2 \lambda _{0}^2}}{s^2-c^2}, \quad \lambda _{2}=\frac{-c(\lambda _{0}-\alpha _{0})-\sqrt{\alpha _{0}^2 s^2-2 \lambda _{0} \alpha _{0}s^2+c^2 \lambda _{0}^2}}{s^2-c^2},  \\ C_{1}&= \frac{N(0)-\gamma _{4} J(0)}{\gamma _{3}-\gamma _{4}}, \quad \gamma _{3}=\left( c \lambda _{0}+\sqrt{s^2 \alpha _{0}^2-2\alpha _{0} \lambda _{0}s^2+c^2 \lambda _{0}^2}\right) /(\alpha _{0} s^2), \end{aligned}$$

65 $$C_{2}=  \frac{\gamma _{3} J(0)-N(0)}{\gamma _{3}-\gamma _{4}}, \quad \gamma _{4}=\left( c \lambda _{0}-\sqrt{s^2 \alpha _{0}^2-2\alpha _{0} \lambda _{0}s^2+c^2 \lambda _{0}^2}\right) /(\alpha _{0} s^2). $$

For $\alpha _{0}<2\lambda _{0}$, $\lambda _{1}$ and $\lambda _{2}$ have the same sign (we note that $\lambda _{1}\lambda _{2}=-\alpha _{0} (\alpha _{0}-2\lambda _{0})/(s^2-c^2)$). Then $S_{1} \notin Y_{S}$, because bounded solutions will be represented only in a half plane.

**Fig. 3:**
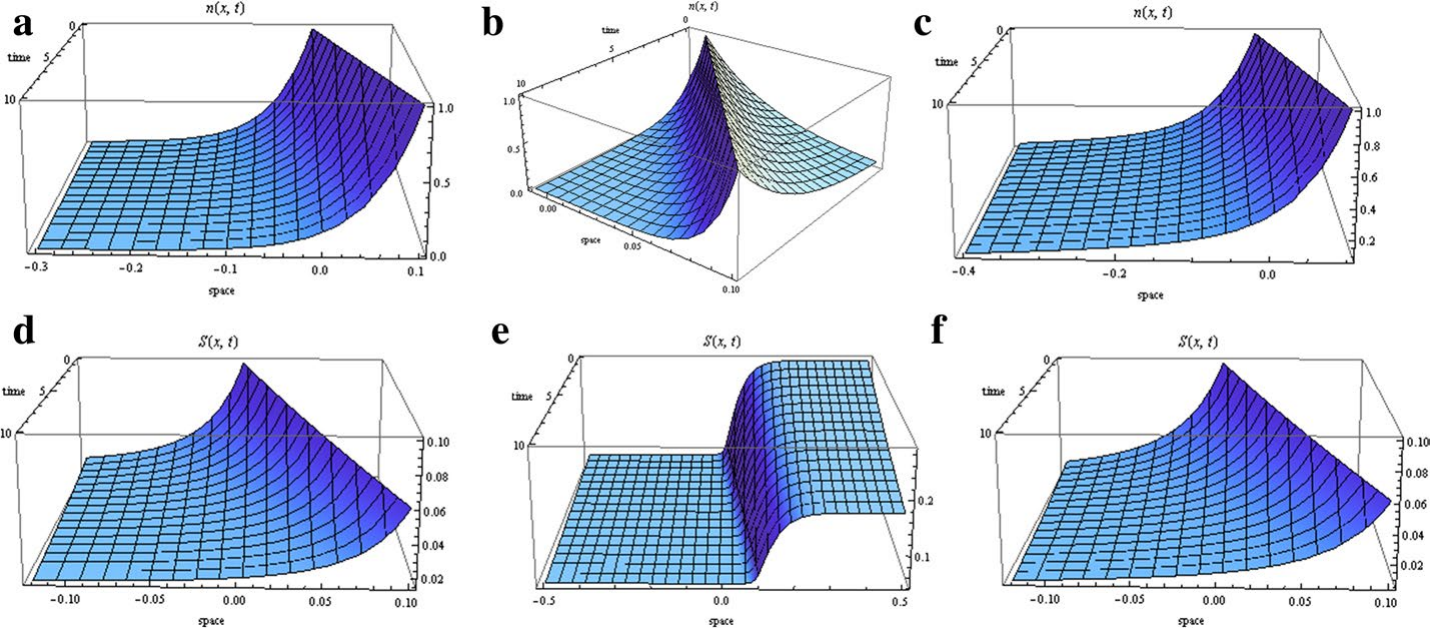
Distribution of cells (*first row*) and non-diffusing signal (*second row*) in the absence of chemotaxis (with the parameters set as follows: $c=10\,\upmu \hbox {m/s}$, $c_{2}=-\gamma =-0.05/\hbox {s}$, $N(0)=1$ and $S_{1}(0)=0.1$). **a** ($\alpha _{0}=0.6$, $\lambda _{0}=0.3$), **b** ($\alpha _{0}=0.9$, $\lambda _{0}=0.1$), **c** ($\alpha _{0}=0.6$, $\lambda _{0}=0.31$), **d** ($\alpha _{0}=0.6$, $\lambda _{0}=0.3$), **e** ($\alpha _{0}=0.9$, $\lambda _{0}=0.1$), **f** ($\alpha _{0}=0.6$, $\lambda _{0}=0.31$)

When $\alpha _{0}>2\lambda _{0}$, then $\lambda _{1}>0$ and $\lambda _{2}<0$. We will choose $C_{1}$ and $C_{2}$ so that *N*(*u*) will not blow up as $u \rightarrow \pm \infty$. For $u<0$, we will take $C_{2}=0$, and for $u\ge 0$, we will take $C_{1}=0$. This will require discontinuity of the flux at zero. Non-diffusing travelling wave solutions (admitting a single peak of *S*) do not exist. However, the requirement that $S_{1} \in Y_{S}$ is less important in the case of no chemotaxis. We remark that Xue et al. ’s (2011) results reflected this relaxation. In our case we do find travelling wave solutions (see Fig. 3).

##### **Theorem 3**

*Non-diffusing* ($D_{S}= 0$) *generalized travelling wave solutions for the system* (11–13) *exist without the restriction* $S_{1}$ *holding in* $Y_{S}$ (*with* $\chi =0$). *They are explicitly given by*

66 $$n(x,t)=N(u)= N(0) \hbox{e}^{(c \alpha _{0}/(s^2-c^2)) u}, \quad j(x,t)=J(u)=J(0)-\frac{s^2}{c} N(0)\left( 1- \hbox{e}^{(c \alpha _{0}/(s^2-c^2)) u} \right),$$

*and* $$S(x,t)=S_{1}(u)\hbox{e}^{c_{2} t}$$, *where*

67 $$S_{1}(u)= S_{1}(0) \exp \left( \frac{\alpha N(0)(s^2-c^2)}{c^2 \alpha _{0}}(\hbox{e}^{(c \alpha _{0}/(s^2-c^2)) u}-1)+\frac{\gamma +c_{2}}{c}u \right) ,$$

*for* $\alpha _{0}=2\lambda _{0}$ (*with* $- \gamma \le c_{2}<0$ *and* $u\le 0$). *In the case of* $\alpha _{0}\ne 2\lambda _{0}$, *the solutions are given by*

68 $$n(x,t)=N(u)= {\left\{ \begin{array}{ll} N(0) \hbox{e}^{\lambda _{1} u}, &{\quad} u< 0, \\ N(0) \hbox{e}^{\lambda _{2} u}, &{\quad} u \ge 0, \end{array}\right. } \quad j(x,t)=J(u)= {\left\{ \begin{array}{ll} \frac{N(0)}{\gamma _{3}} \hbox{e}^{\lambda _{1} u}, &{\quad} u < 0, \\ \frac{N(0)}{\gamma _{4}} \hbox{e}^{\lambda _{2} u}, &{\quad} u \ge 0, \end{array}\right. }$$

*and* $$S(x,t)=S_{1}(u)\hbox{e}^{c_{2} t}$$, *where*

69 $$S_{1}(u)= {\left\{ \begin{array}{ll} S_{1}(0) \exp \left( \frac{\alpha N(0)}{c \lambda _{1}}(\hbox{e}^{\lambda _{1}u}-1) \right) , &{\quad} u < 0, \\ S_{1}(0) \exp \left( \frac{\alpha N(0)}{c \lambda _{2}}(\hbox{e}^{\lambda _{2}u}-1) \right) , &{\quad} u \ge 0, \end{array}\right. } $$

*for* $\alpha _{0}>2\lambda _{0}$ (*with* $c_{2}=-\gamma$), *and by* (63) *and* $S(x,t)=S_{1}(u)\hbox{e}^{c_{2} t}$, *where*

70 $$S_{1}(u)= S_{1}(0) \exp \left( \frac{\alpha \gamma _{3} C_{1}}{c \lambda _{1}}(\hbox{e}^{\lambda _{1} u}-1)+\frac{\alpha \gamma _{4} C_{2}}{c \lambda _{2}}(\hbox{e}^{\lambda _{2} u}-1)+\frac{\gamma +c_{2}}{c}u \right) , $$

*for* $\alpha _{0}<2\lambda _{0}$ (*with* $u\le 0$ *and* $-\gamma \le c_{2}<0$, *if* $\lambda _{1}>0$, *or* $u\ge 0$ *and* $c_{2}=-\gamma$, *if* $\lambda _{1}<0$).□

The non-diffusing solutions $S_{1}(u)$ of Theorem 3 are obtained directly by integrating the first order system (59–61). We note that all of the solutions are bounded, for they are continuous and converge at the boundaries. A negative flux (see (66)) simply means that most of the cells move to the left (recall that $j=s(n^{+}-n^{-})$). Unlike Franz et al. ’s (2014) results, we do not require a minimal wave speed. This therefore constitutes a generalization of their findings.

We note that the discontinuity of the flux at zero does not necessarily imply *N*(*u*) to be discontinuous at zero. In fact, for $J(u)=sN(u)$ and the initial conditions given by $N^{+}(0^{+})>N^{+}(0^{-})$, $N^{-}(0^{-})=N^{+}(0^{+})$ and $N^{-}(0^{+})=N^{+}(0^{-})$, we have

71 $$N(0^{+})-N(0^{-})=N^{+}(0^{+})+N^{-}(0^{+})-N^{+}(0^{-})-N^{-}(0^{-})=0,$$

and

72 $$J(0^{+})\,-\,J(0^{-})=s(N^{+}(0^{+})\,-\,N^{-}(0^{+})\,-\,N^{+}(0^{-})+N^{-}(0^{-}))=2s(N^{+}(0^{+})\,-\,N^{+}(0^{-}))\ne 0. $$

##### **Theorem 4**

*For* $D_{S} \ne 0$, $\alpha _{0}>2\lambda _{0}$, $-\gamma <c_{2}\le 0$ *and* $\gamma _{3}J(0^{-})=\gamma _{4}J(0^{+})=N(0)$, *travelling wave solutions (with* $S_{1} \in Y_{S}$) *for the system* (11–13) *exist and are explicitly given by*

73 $$n(x,t)=N(u)= {\left\{ \begin{array}{ll} N(0) \hbox{e}^{\lambda _{1} u}, & u< 0, \\ N(0) \hbox{e}^{\lambda _{2} u}, & u \ge 0, \end{array}\right. } \quad j(x,t)=J(u)= {\left\{ \begin{array}{ll} \frac{N(0)}{\gamma _{3}} \hbox{e}^{\lambda _{1} u}, & u < 0, \\ \frac{N(0)}{\gamma _{4}} \hbox{e}^{\lambda _{2} u}, & u \ge 0, \end{array}\right. } $$

*and* $$S(x,t)=S_{1}(u)\hbox{e}^{c_{2}t}$$, *where*

74 $$S_{1}(u)= {\left\{ \begin{array}{ll} c_{1}^{1} I_{k_{1}}\left( \alpha _{1} \hbox{e}^{(\lambda _{1}/2)u} \right) \hbox{e}^{-(c/(2D_{S})) u}, & u < 0, \\ c_{1}^{2}I_{k_{2}}\left( \alpha _{2} \hbox{e}^{(\lambda _{2}/2)u} \right) \hbox{e}^{-(c/(2D_{S})) u}, & u \ge 0, \end{array}\right. }$$

*with* $$u=x-ct$$, $$k_{1}=(\sqrt{c^2+4D_{S}(\gamma +c_{2})}/(\lambda _{1} D_{S})$$, $$k_{2}=-\sqrt{c^2+4D_{S}(\gamma +c_{2})}/(\lambda _{2} D_{S})$$, $$\alpha _{1}=\sqrt{4 \alpha D_{S}N(0)}/(\lambda _{1} D_{S})$$, $$\alpha _{2}=-\sqrt{4 \alpha D_{S}N(0)}/(\lambda _{2} D_{S})$$, *and* $$c_{1}^{1} I_{k_{1}}(\alpha _{1})=c_{1}^{2}I_{k_{2}}(\alpha _{2})=S_{1}(0)$$.

**Fig. 4:**
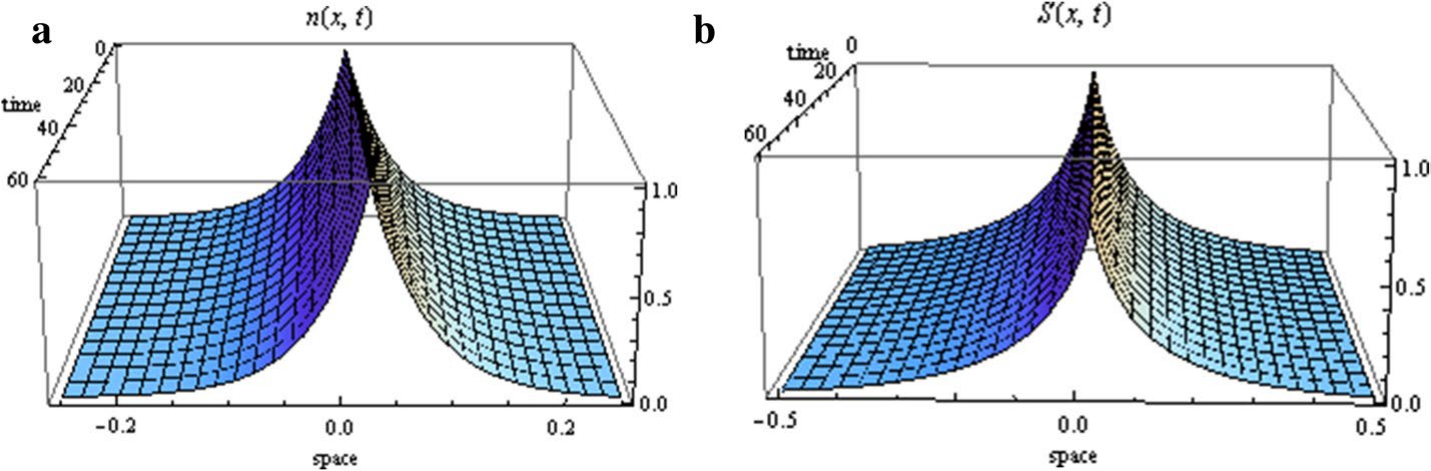
Spread of cells (**a**) and signal (**b**) in the case of no chemotaxis under growth ($\lambda _{0}=0.3$, $\alpha _{0}=0.8$, $N(0)=1$ and $S_{1}(0)=1$)

The proof of the above theorem is similar to the proof of Theorem 1. See Fig. 4 for an illustration of the solutions.

#### High chemotactic sensitivity ($k \rightarrow 0$)

Here $\lambda ^{1}$ is given by (14). For $S_{1} \in Y_{S}$, the system (40–42) becomes

75 $$ N^{\prime}= a_{1} N+b_{1} J,$$

76 $$J^{\prime}=   a_{2} N+cb_{1} J,$$

77 $$-c S^{\prime}_{1}=  D_{S} S^{\prime\prime}_{1} -(\alpha N+\gamma +c_{2}) S_{1}, $$

where

78 $$a_{1}=\frac{c \alpha _{0}-2 \lambda _{0} s}{s^2-c^2}, \quad b_{1}=\frac{\alpha _{0}-2\lambda _{0}}{s^2-c^2}, \quad a_{2}=\frac{s^2 \alpha _{0}-2 \lambda _{0} cs}{s^2-c^2}, $$

for $$u>0$$, and

79 $$a_{1}=\frac{c \alpha _{0}+2 \lambda _{0} s}{s^2-c^2}, \quad b_{1}=\frac{\alpha _{0}-2\lambda _{0}}{s^2-c^2}, \quad a_{2}=\frac{s^2 \alpha _{0}+2 \lambda _{0} cs}{s^2-c^2}, $$

for $$u<0$$.

When $\alpha _{0}=2 \lambda _{0}$, the coefficient $b_{1}$ vanishes and we obtain

80 $$N(u)= {\left\{ \begin{array}{ll} N(0) \hbox{e}^{\frac{\alpha _{0}}{s-c}u}, &{\quad} u< 0, \\ N(0) \hbox{e}^{-\frac{\alpha _{0}}{s+c}u}, &{\quad} u \ge 0, \end{array}\right. } \quad J(u)= {\left\{ \begin{array}{ll} J(0)-sN(0) \left( 1- \hbox{e}^{\frac{\alpha _{0}}{s-c}u}\right) , &{\quad} u < 0, \\ J(0)+sN(0) \left( 1- \hbox{e}^{-\frac{\alpha _{0}}{s+c}u}\right) , &{\quad} u \ge 0. \end{array}\right. } $$

We note that the total cell population is given by $T_{2}=2sN(0)/(2\lambda _{0}-\alpha _{0})$, and the flux can be negative. This simply means that most of the cells move to the left [we recall that $j=s(n^{+}-n^{-})$]. The analysis in this situation is mathematically similar to the case of zero growth in Sect. 2.2. Travelling wave solutions satisfying $S_{1} \in Y_{S}$ exist only in the case of diffusivity, underlying the importance of introducing this biological process into the model.

##### **Theorem 5**

*For* $D_{S} \ne 0$, $\alpha _{0}=2\lambda _{0}$ *and* $-\gamma <c_{2}\le 0$, *travelling wave solutions for the system* (11–13) *exist and are given by* (80) *and* $S(x,t)=S_{1}(u)\hbox{e}^{c_{2}t}$, *where*

81 $$S_{1}(u)= {\left\{ \begin{array}{ll} c_{1}^{1} I_{k_{1}}\left( \alpha _{1} \exp \left( \frac{\alpha _{0}}{2(s-c)} u\right) \right) \hbox{e}^{-(c/(2D_{S})) u}, & u < 0, \\ c_{1}^{2}I_{k_{2}}\left( \alpha _{2} \exp \left( - \frac{\alpha _{0}}{2(s+c)} u\right) \right) \hbox{e}^{-(c/(2D_{S})) u} , & u \ge 0, \end{array}\right. }$$

*with* $u=x-ct$, $k_{1}=(s-c)\sqrt{c^2+4D_{S}(\gamma +c_{2})}/(\alpha _{0}D_{S})$, $k_{2}=(s+c)\sqrt{c^2+4D_{S}(\gamma +c_{2})}/(\alpha _{0}D_{S})$, $\alpha _{1}=(s-c)\sqrt{4 \alpha D_{S}N(0)}/(\alpha _{0}D_{S})$, $\alpha _{2}=(s+c)\sqrt{4 \alpha D_{S}N(0)}/(\alpha _{0}D_{S})$, *and* $c_{1}^{1} I_{k_{1}}(\alpha _{1})=S_{1}(0)$ *and* $c_{1}^{2}I_{k_{2}}(\alpha _{2})=S_{1}(0)$.□

**Fig. 5:**
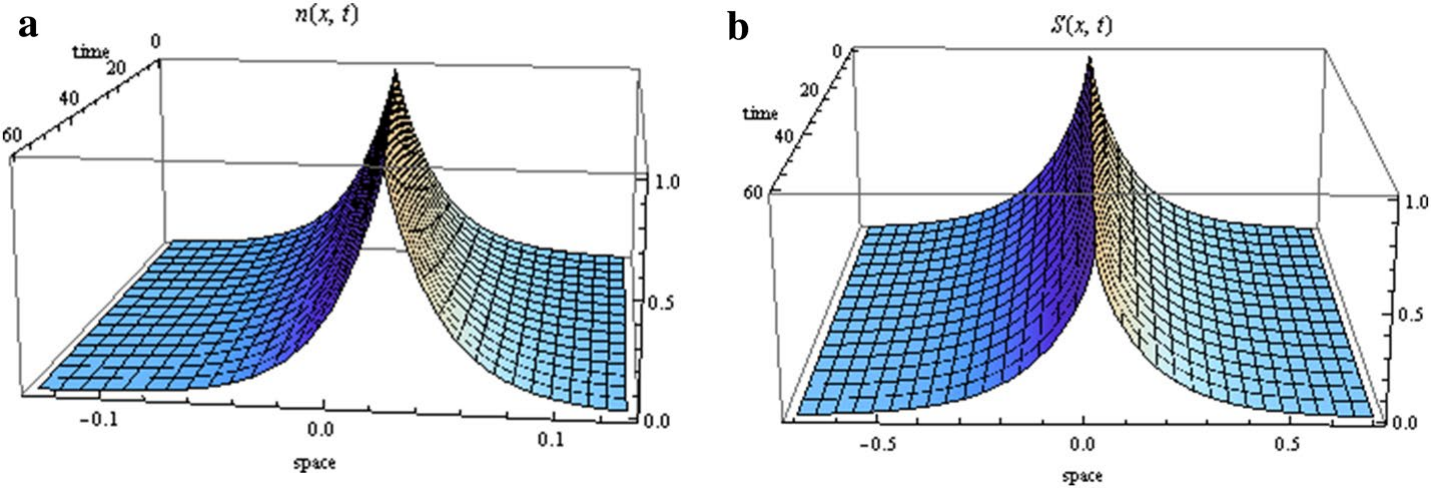
Spread of cells (**a**) and signal (**b**) in the case of high chemotactic sensitivity (with $\lambda _{0}=0.4$ and $\alpha _{0}=0.8$, $N(0)=1$ and $S_{1}(0)=1$)

The proof of the above theorem is similar to the proof of Theorem 1. The solutions are depicted in Fig. 5.

Now we assume $\alpha _{0} \ne 2 \lambda _{0}$, then

82 $$N(u)= {\left\{ \begin{array}{ll} \frac{C_{1}}{s} \hbox{e}^{\lambda _{1}u}+\gamma _{0} C_{2} \hbox{e}^{\lambda _{2}u}, &{\quad} u< 0, \\ -\frac{C_{3}}{s} \hbox{e}^{-\lambda _{3}u}+\gamma _{1} C_{4} \hbox{e}^{-\lambda _{4}u}, &{\quad} u \ge 0, \end{array}\right. } \quad J(u)= {\left\{ \begin{array}{ll} C_{1} \hbox{e}^{\lambda _{1}u}+ C_{2} \hbox{e}^{\lambda _{2}u}, &{\quad} u < 0, \\ C_{3} \hbox{e}^{-\lambda _{3}u}+ C_{4} \hbox{e}^{-\lambda _{4}u}, &{\quad} u \ge 0, \end{array}\right. }$$

where

83 $$\lambda _{1}= \frac{\alpha _{0}}{s-c}, \quad\lambda _{2}=\frac{2 \lambda _{0}-\alpha _{0}}{s+c}, \quad\lambda _{3}=\frac{\alpha _{0}}{s+c}, \quad\lambda _{4}=\frac{2 \lambda _{0}-\alpha _{0}}{s-c}, \quad\gamma _{0}=\frac{2 \lambda _{0}-\alpha _{0}}{s \alpha _{0}+2 c \lambda _{0}}, $$

84 $$ \gamma _{1}= \frac{2 \lambda _{0}-\alpha _{0}}{2 \lambda _{0} c-s \alpha _{0}},\quad C_{1}=\frac{s(\gamma _{0} J(0^{-})-N(0))}{s \gamma _{0}-1},\quad C_{2}=\frac{sN(0)-J(0^{-})}{s \gamma _{0}-1},$$

85 $$C_{3}=  \frac{s(\gamma _{1} J(0^{+})-N(0))}{s \gamma _{1}+1},\quad C_{4}=\frac{sN(0)+J(0^{+})}{s \gamma _{1}+1}. $$

When $C_{1}$ (or $C_{2}$) and $C_{3}$ (or $C_{4}$) are zero, travelling wave solutions are possible.

##### **Theorem 6**

*For* $D_{S} \ne 0$, $\alpha _{0}<2\lambda _{0}$, $-\gamma <c_{2}\le 0$ *and* $\gamma _{0}J(0^{-})=\gamma _{1}J(0^{+})=N(0)$, *travelling wave solutions for the system* (11–13) *exist and are explicitly given by*

86 $$n(x,t)=N(u)= {\left\{ \begin{array}{ll} N(0) \hbox{e}^{\lambda _{2} u}, & u< 0, \\ N(0) \hbox{e}^{-\lambda _{4} u}, & u \ge 0, \end{array}\right. } \quad j(x,t)=J(u)= {\left\{ \begin{array}{ll} \frac{N(0)}{\gamma _{0}} \hbox{e}^{\lambda _{2} u}, & u < 0, \\ \frac{N(0)}{\gamma _{1}} \hbox{e}^{-\lambda _{4} u}, & u \ge 0, \end{array}\right. } $$

*and* $$S(x,t)=S_{1}(u)\hbox{e}^{c_{2}t}$$, *where*

87 $$S_{1}(u)= {\left\{ \begin{array}{ll} c_{1}^{1} I_{k_{1}}\left( \alpha _{1} \hbox{e}^{(\lambda _{2}/2)u} \right) \hbox{e}^{-(c/(2D_{S})) u}, & u < 0, \\ c_{1}^{2}I_{k_{2}}\left( \alpha _{2} \hbox{e}^{-(\lambda _{4}/2)u} \right) \hbox{e}^{-(c/(2D_{S})) u}, & u \ge 0, \end{array}\right. } $$

*with* $$u=x-ct$$, $$k_{1}=(\sqrt{c^2+4D_{S}(\gamma +c_{2})}/(\lambda _{2} D_{S})$$, $$k_{2}=\sqrt{c^2+4D_{S}(\gamma +c_{2})}/(\lambda _{4} D_{S})$$, $$\alpha _{1}=\sqrt{4 \alpha D_{S}N(0)}/(\lambda _{2} D_{S})$$, $$\alpha _{2}=\sqrt{4 \alpha D_{S}N(0)}/(\lambda _{4} D_{S})$$, *and* $$c_{1}^{1} I_{k_{1}}(\alpha _{1})=c_{1}^{2}I_{k_{2}}(\alpha _{2})=S_{1}(0)$$.□

**Fig. 6:**
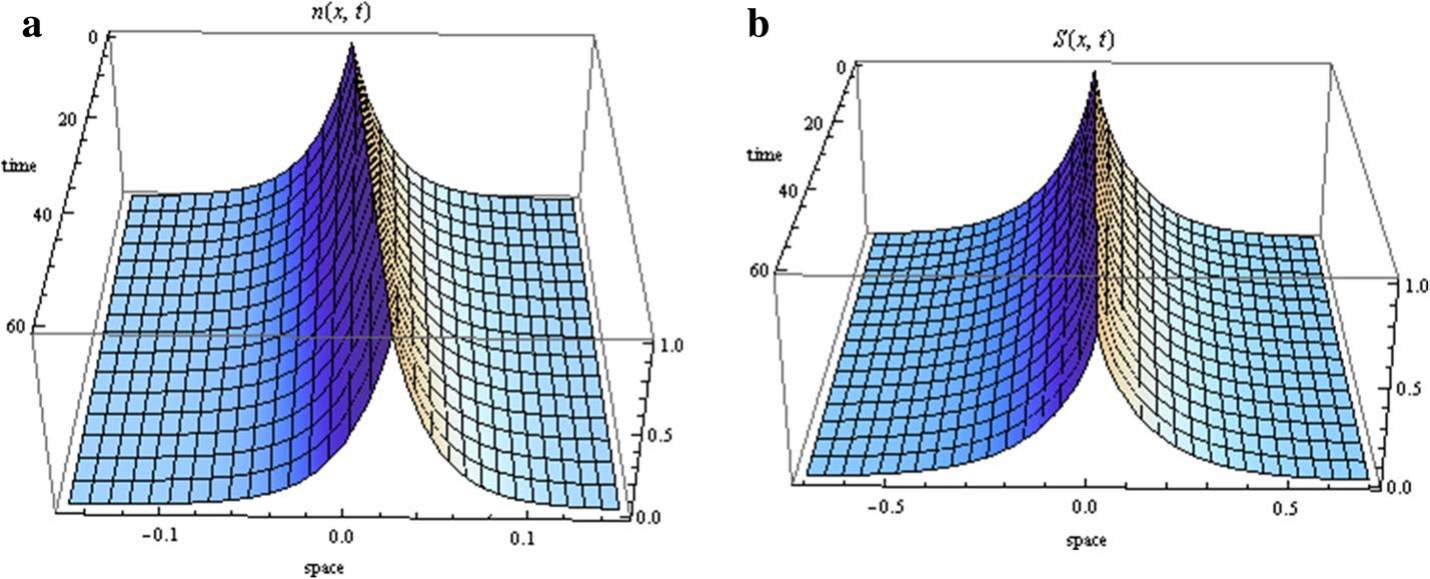
Spread of cells and signal in the case of unbounded sensitivity to the signal, with $\alpha_{0}< 2 \lambda_{0} \, (\lambda_{0}= \alpha_{0} = 0.8,\, N(0) = 1 \, {\text{and}} \, S_{1}(0) = 1)$ . The sub-figure **a** depicts the distribution of the cells and the sub-figure **b** depicts the distribution of the substrates

In the above theorem, the total cell population is given by $T_{3}=2N(0)/\alpha _{0}$. We illustrate the solutions in Fig. 6.

When at most one of the constants $C_{i}$ is zero (for instance $C_{2}$), Eq. (77) is difficult to solve explicitly for $S_{1}(u)$ (given the form of *N*(*u*) when $u>0$). In this situation, we only look at the asymptotic behaviour of the solutions as $u\rightarrow \pm \infty$. We note that the origin is the only equilibrium point of the system (75–77), and the determinant of the corresponding Jacobian matrix around the origin is given by

88 $${\Delta } =\frac{\alpha _{0}(c_{2}+\gamma )(\alpha _{0}-2\lambda _{0})}{D_{S}(s^2-c^2)}.$$

We consider $\alpha _{0}<2\lambda _{0}$ to guarantee the stability of *N*(*u*) and *J*(*u*)(see (83)). The eigenvalues dictating the behaviour of $S_{1}(u)$ are given by

89 $$\lambda _{11}=\frac{-c+\sqrt{c^2+4D_{S}(c_{2}+\gamma )}}{2D_{S}}, \quad \hbox {and} \quad \lambda _{22}=-\frac{c+\sqrt{c^2+4D_{S}(c_{2}+\gamma )}}{2D_{S}}. $$

**Fig. 7:**
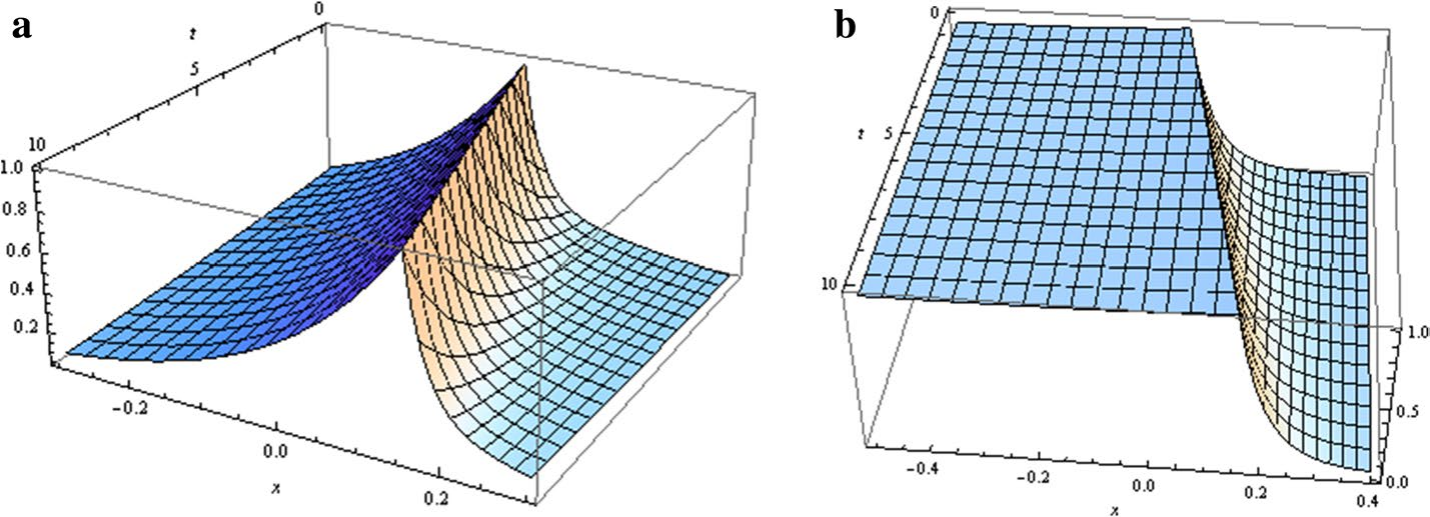
Spread of cells and signal in the case of unbounded sensitivity to the signal, with $\alpha_{0} < 2 \lambda _{0}$ and only $C_1 = 0 \, (\lambda_{0} = 0.2, \, c = 0.015, \, \alpha_{0} = 0.21, \, N(0) = 1, \, S_{1} (0) = 1)$. The sub-figure **a** depicts the distribution of the cells and the sub-figure **b** depicts the distribution of the substrates

For $c_{2}+\gamma <0$, $\lambda _{11}$ and $\lambda _{22}$ are both negative; non growing solutions are possible only in the half plane $u\ge 0$. Then $S_{1}$ cannot hold in $Y_{S}$. For $c_{2}+\gamma >0$, $\lambda _{11}$ and $\lambda _{22}$ have opposite signs. To obtain convergence, we will choose the initial data so that the behaviour of $S_{1}(u)$ will be controlled only by $\lambda _{22}$ and $\lambda _{11}$ as *u* approaches $+\infty$ and $-\infty$, respectively (this method was also applied in §2.3.1). In this situation $S_{1}(u)$ is positive, since none of the eigenvalues is complex (We note that the nonlinear term does not affect the stability of $S_{1}$, given that the eigenvalues are nonzero.). The challenge in getting explicit solutions for $S_{1}(u)$ prevents us from checking whether the restriction $S_{1} \in Y_{S}$ holds for all real *u* or not. However, this restriction can be guaranteed as $u \rightarrow \pm \infty$. In fact, from (82), as $u\rightarrow \pm \infty$,

90 $$N(u) \approx {\left\{ \begin{array}{ll} \delta _{1} \hbox{e}^{\mu _{1} u}, & \quad  u < 0, \\ \delta _{2} \hbox{e}^{-\mu _{2} u}, & \quad u > 0, \end{array}\right. }$$

where $\mu _{1}=\min (\lambda _{1},\lambda _{2})$, $\mu _{2}=\min (\lambda _{3},\lambda _{4})$, and $\delta _{1}$, $\delta _{2}$ are coefficients of the dominant terms $\hbox{e}^{\mu _{1}u}$ and $\hbox{e}^{-\mu _{2}u}$. Substituting (90) into (77) then integrating, and taking into consideration the boundedness conditions, one obtains, as $u\rightarrow \pm \infty$,

91 $$S_{1}(u)\approx {\left\{ \begin{array}{ll} c_{1}^{1} I_{k_{1}}\left( \alpha _{1} \hbox{e}^{(\mu _{1}/2)u} \right) \hbox{e}^{-(c/(2D_{S})) u}, & \quad u < 0, \\ c_{1}^{2}I_{k_{2}}\left( \alpha _{2} \hbox{e}^{-(\mu _{2}/2)u} \right) \hbox{e}^{-(c/(2D_{S})) u}, & \quad u > 0, \end{array}\right. } $$

where $k_{i}=(\sqrt{c^2+4D_{S}(\gamma +c_{2})}/(\mu _{i} D_{S})$, $\alpha _{i}=\sqrt{4 \alpha D_{S}\delta _{i}}/(\mu _{i} D_{S})$, and $c_{1}^{1}$ and $c_{1}^{2}$ are positive constants. We previously proved that $S_{1}\in Y_{S}$. Asymptotic travelling wave solutions are possible. The asymptotic behaviour of $S_{1}(u)$ is illustrated in Fig. 7.

## Discussion

In this paper we studied the existence of travelling wave solutions (with a single peak for the signal) of a microscopic model for chemotaxis. We focused on the case of starvation; cells in this situation consume signal only. The effect of microscale parameters in the stability of the system was examined. Unlike previous approaches, we allowed for degradation of signal ($\gamma \ne 0$). While we will compare our results to those previously obtained, it must be borne in mind that this important biological process was not considered in other results. We performed a Lie symmetry analysis to generate a large class of invariants leading to generalized travelling wave solutions. Only relevant invariants were considered, but we believe that rich information could have been extracted from the full form of invariants in different contexts. We provided explicit solutions, many for the first time.

We first considered the case of zero growth. Such a scenario is possible if the time interval is shorter than the period required for cell proliferation. When we imposed no chemotaxis, we could not find travelling wave solutions satisfying $S_{1} \in Y_{S}$. We note that Xue et al. (2011) also indicated the absence of travelling wave solutions when there is no chemotaxis. However, their results held in the case of no diffusivity ($D_{S} =0$). We have shown here that these results also hold in the diffusing case. However, when we relaxed the restriction on $S_{1}$ (less important in the absence of chemotaxis), we obtained both diffusing and non-diffusing travelling wave solutions, distributed in a half plane (see Fig. 2).

If we now consider the high chemotactic limit, we find that non-diffusing ($D_{S}=0$) travelling wave solutions do not exist. This is in contrast to Xue et al. ’s (2011) results in which they were found to exist. The degradation of the signal removed this possibility in our results. However, as evidenced in Theorem 1, the incorporation of diffusion does allow for the existence of travelling wave solutions. It is interesting to note that diffusion, in a sense, counteracts the wave eradication effect of the degradation of the signal.

In order to model the behaviour of cells over more realistic time frames, we incorporated cell growth into our model. As a first attempt we assumed constant growth. In general, with no chemotaxis, non-diffusing ($D_{S}= 0$) travelling wave solutions do not exist. However, we observe that this occurs due to the requirement that $S_{1} \in Y_{S}$. This is not necessary in the case of no chemotaxis. Relaxing this restriction leads to non-diffusing travelling wave solutions (see Theorem 3). Note that, unlike Xue et al. (2011), we dot not require a minimal wave speed. We are also able to find diffusing travelling wave solutions (with a discontinuous flux) provided the growth rate dominates the dynamics (see Theorem 4).

If we consider the high chemotactic limit we find that non-diffusing travelling wave solutions do not exist. However, incorporating diffusivity leads to the possibility of travelling wave solutions (see Theorems 5 and 6 ). Note that these are the first results in the case of high chemotaxis with non-zero growth. In contrast to Keller and Segel’s (1970, 1971a) results in the macroscopic model under zero growth, none of our travelling wave solutions required a singularity in the chemotactic sensitivity.

When cells are highly sensitive to signals, allowing for diffusivity, we observe for $\alpha _{0}<2\lambda _{0}$ that the total cell population (given by $T_{2}=2sN(0)/(2\lambda _{0}-\alpha _{0})$) increases as the growth rate $\alpha _{0}$ becomes large; here most of the new born cells remain in the band. For $\alpha _{0}=2\lambda _{0}$, the growth rate controls the behaviour of the system (see § 2.3.2). In this case, the total cell population (given by $T_{3}=2sN(0)/\alpha _{0}$) decreases as $\alpha _{0}$ is large. This is due to the local depletion of the signal; some cells will move towards regions with higher concentrations of signal (this is typical in chemotactic systems). The aggregated cells here do not disperse as we demonstrated the existence of travelling wave solutions in this situation. However, for $\alpha _{0}>2\lambda _{0}$, we notice instability. The cell growth rate controls the behaviour of the system, and prevents formation of the aggregation.

The inverse phenomenon is observed in the limiting case where cells are not sensitive to the signal gradient; they move randomly in this situation. For $\alpha _{0} \le 2\lambda _{0}$, we obtained instability in the system, travelling wave solutions do not exist. However, for $\alpha _{0}>2\lambda _{0}$, the stability of the solutions is controlled by the growth rate $\alpha _{0}$. We imposed restrictions on the initial conditions in order to foster a collective behaviour. Travelling wave solutions then resulted. This result is in agreement with Lauffenburger et al. ’s (1984) findings (in the macroscopic model), in which travelling wave solutions exist due to the balance of growth, death and random motility.

As result of our investigation, we remark that cell growth and cell unbiased turning rate play an important role in the stability of the system and the aggregation of cells. We also remark that the total cell population in the case of zero growth ($T_{1}$) is less than that of the case of constant growth ($T_{2}$ and $T_{3}$). The wider band of cells is obtained in the case of no chemotaxis (we recall that here the growth rate $\alpha _{0}$ controls the stability of the system, the absence of sensitivity to stimuli keep most of cells in the band). The distribution of cells are displayed in Figs. 1a, 2a, 3a, d, 4a, 5a and 6a. We also note that the total cell population $T_{i}$ decreases as $\lambda _{0}$ becomes large; the permanent change of direction does not necessarily destabilize the formation of bands of bacteria.

## Conclusion

We have shown that it is crucial to consider the individual response of cells when studying their macroscopic behaviour. This helps us to capture microscale information which play a significant role in the system. Our results can be summarized in Table 1. For future work, we will consider a higher dimensional space and will investigate the geometric shape of bands of bacteria.

**Table 1:** Table summarizing our findings on the existence of travelling wave solutions (TWS)

*h*(*S*)	*k*	$D_{S}$	TWS	Restriction on $\alpha _{0}$
0	$\infty$	0	$\times$ (or $\surd$ if $S_{1} \notin Y_{S}$)	–
		$\ne 0$	$\times$ (or $\surd$ if $S_{1} \notin Y_{S}$)	–
	0	0	$\times$	–
		$\ne 0$	$\surd$	–
$\alpha _{0}$	$\infty$	0	$\times$ (or $\surd$ if $S_{1} \notin Y_{S}$)	– (or arbitrary)
		$\ne 0$	$\surd$	$\alpha _{0}>2 \lambda _{0}$
	0	0	$\times$	–
		$\ne 0$	$\surd$	$\alpha _{0}\le 2 \lambda _{0}$

## References

[CR1] Adler J (1966). Chemotaxis in bacteria. Science.

[CR2] Adler J (1966). Effect of amino acids and oxygen on chemotaxis in *Escherichia coli*. J Bacteriol.

[CR3] Adler J (1975). Chemotaxis in bacteria. Annu Rev Biochem.

[CR4] Alt W (1980). Biased random walk models for chemotaxis and related diffusion approximations. J Math Biol.

[CR5] Bak B, Tang C, Wiesenfeld K (1987). Self-organized criticality: an explanation of 1/*f* noise. Phys Rev Lett.

[CR6] Berg JM, Tymoczko JL, Stryer L (2002) Biochemistry, 5th edn. WH Freeman, New York

[CR7] Beyerinck M (1895). Ueber Spirillum desulfuricans als ursache von sulfatreduction. Zentralbl Bakteriol Parasitenkd.

[CR8] Blat Y, Eisenbach MJ (1995). Tar-dependent and independent pattern formation by salmonella typhimurium. Bacteriology.

[CR9] Bluman GW, Anco SC (2002). Symmetry and integration methods for differential equations.

[CR10] Brenner M, Levitor L, Brudrene E (1998). Physical mechanisms for chemotactic pattern formation by bacteria. Biophys J.

[CR11] Budrene EO, Berg HC (1991). Complex patterns formed by motile cells of *Escherichia coli*. Nature.

[CR12] Budrene EO, Berg HC (1995). Dynamics of formation of symmetrical patterns by chemotactic bacteria. Nature.

[CR14] Clarkson PA (1995). Nonclassical symmetry reductions for the Boussinesq equation. Chaos Solitons Fractals.

[CR15] Condeelis JS, Wyckoff JB, Bailly M, Pestell R, Lawrence D, Backer J, Segall JE (2001). Lamellipodia in invasion. Semin Cancer Biol.

[CR16] Devreotes P, Janetopoulos C (2003). Eukaryotic chemotaxis: distinctions between directional sensing and polarization. J Biol Chem.

[CR17] Eisenbach M, Lengeler JW (2004). Chemotaxis.

[CR18] Engelmann T (1881). Neue methode zur untersuchung der sauerstoffaussheidung pflanzlicher und thierischer organismen. Pflugers Arch Gesamte Physiol.

[CR19] Engelmann T (1881). Zur biologie der schizomycete. Pflugers Arch Gesamte Physiol.

[CR20] Entschladen F, Zanker KS (2002). Cell migration: signalling and mechanisms. Transl Res Biomed Basel.

[CR21] Friedrich BM, Jülicher F (2007). Chemotaxis of sperm cells. Proc Natl Acad Sci USA.

[CR22] Franz B, Xue C, Painter K, Erban R (2014). Travelling waves in hybrid chemotaxis models. Bull Math Biol.

[CR23] Gangur V, Birmingham NP, Thanesvorakul S (2002). Chemokines in health and disease. Vet Immunol Immunopathol.

[CR25] Hillen T, Painter KJ (2009). A users guide to PDE models for chemotaxis. J Math Biol.

[CR26] Keller EF, Segel LA (1970). Initiation of slim mold aggregation viewed as an instability. J Theor Biol.

[CR27] Keller EF, Segel LA (1971). Model for chemotaxis. J Theor Biol.

[CR28] Keller EF, Segel LA (1971). Travelling bands of chemotactic bacteria: a theoretical analysis. J Theor Biol.

[CR29] Lajkó E, Szabó I, Andódy K, Pungor A, Mezö G, Köhidai L (2013). Investigation on chemotactic drug targeting (chemotaxis and adhesion) inducer effect of GnRH-III derivatives in Tetrahymena and human leukemia cell line. J Pept Sci.

[CR30] Lauffenburger DA, Kennedy C, Aris R (1984). Travelling band of bacteria in the context of population growth. Bull Math Biol.

[CR31] Lui R, Wang ZA (2010). Travelling wave solutions from microscopic to macroscopic chemotaxis models. J Math Biol.

[CR32] Maki N, Gestwicki JE, Lake EM, Kiessling LL, Adler J (2000). Motility and chemotaxis of filamentous cells of *Escherichia coli*. J Bacteriol.

[CR33] McLachlan NW (1955). Bessel functions for engineers.

[CR34] Moore MA (2001). The role of chemoattraction in cancer metastases. Bioessays.

[CR35] Murphy PM (2001). Chemokines and the molecular basis of cancer metastasis. N Engl J Med.

[CR36] Murray J (2002). Mathematical biology.

[CR37] Nadin G, Perthame B, Ryzhik L (2008). Traveling waves for the Keller–Segel system with Fisher birth term. Interfaces Free Bound.

[CR38] Olver FW, Lozier DW, Boisvert RF, Clark CW (2010). NIST handbook of mathematical functions.

[CR39] Othmer HG, Dunbar SR, Alt W (1988). Models of dispersal in biological systems. J Math Biol.

[CR40] Patlak CS (1953). Random walk with persistence and external bias. Bull Math Biophys.

[CR41] Paul K, Nieto V, Carlquist WC, Blair DF, Harshey RM (2010). The c-di-GMP binding protein YcgR controls flagellar motor direction and speed to affect chemotaxis by a “Backstop Brake” mechanism. Mol Cell.

[CR42] Pfeffer W (1888). Uber chemotaktische bewegungen von bacterien, flagellaten and volvocineen. Unters Bot Inst Tbingen.

[CR43] Polyanin AD, Zaitsev VF (2004). Handbook of nonlinear partial differential equations.

[CR44] Rosen G (1977). Effects of diffusion on the stability of the equilibrium in multi-species ecological systems. Bull Math Biol.

[CR45] Sahari A, Traore MA, Scharf BE, Behkam B (2014). Directed transport of bacteria-based drug delivery vehicles: bacterial chemotaxis dominates particle shape. Biomed Microdevices.

[CR46] Schneider L, Cammer M, Lehman J, Nielsen SK, Guerra CF, Veland IR, Stock C, Hoffmann EK, Yoder BK, Schwab A, Satir P, Christensen ST (2010). Directional cell migration and chemotaxis in wound healing response to PDGF-AA are coordinated by the primary cilium in fibroblasts. Cell Physiol Biochem.

[CR47] Scribner T, Segel L, Rogers E (1974). A numerical study of the formation and propagation of travelling bands of chemotactic bacteria. J Theor Biol.

[CR49] Tchepmo Djomegni PM, Govinder KS (2014). The interplay of group and dynamical systems analysis: the case of spherically symmetric charged fluids in general relativity. Int J Non-Linear Mech.

[CR50] Tchepmo Djomegni PM, Govinder KS (2016). Generalized travelling wave solutions for hyperbolic chemotaxis PDEs. Appl Math Mod.

[CR51] Tchepmo Djomegni PM, Govinder KS (2015b) Asymptotic analysis of travelling wave solutions in chemotaxis with nutrients dependant cell growth (Preprint: School of Mathematics. University of KwaZulu-Natal, Durban). Statistics and Computer Science

[CR52] Wang ZA (2013). Mathematics of travelling waves in chemotaxis. Discrete Contin Dyn Syst Ser B.

[CR54] Woodward DE, Tyson R, Myerscough MR, Budrene EO, Berg HC (1995). Spacio-temporal patterns generated by *Salmonella typhimurium*. Biophys J.

[CR55] Xue C, Hwang HJ, Painter KJ, Erban E (2011). Travelling waves in hyperbolic chemotaxis equations. Bull Math Biol.

